# Mathematical multi-scale model of the cardiovascular system including mitral valve dynamics. Application to ischemic mitral insufficiency

**DOI:** 10.1186/1475-925X-10-86

**Published:** 2011-09-24

**Authors:** Sabine Paeme, Katherine T Moorhead, J Geoffrey Chase, Bernard Lambermont, Philippe Kolh, Vincent D'orio, Luc Pierard, Marie Moonen, Patrizio Lancellotti, Pierre C Dauby, Thomas Desaive

**Affiliations:** 1 Cardiovascular Research Center, University of Liege, Liege, Belgium; 2 Department of Mechanical Engineering, University of Canterbury, Christchurch, New Zealand

## Abstract

**Background:**

Valve dysfunction is a common cardiovascular pathology. Despite significant clinical research, there is little formal study of how valve dysfunction affects overall circulatory dynamics. Validated models would offer the ability to better understand these dynamics and thus optimize diagnosis, as well as surgical and other interventions.

**Methods:**

A cardiovascular and circulatory system (CVS) model has already been validated *in silico*, and in several animal model studies. It accounts for valve dynamics using Heaviside functions to simulate a physiologically accurate "open on pressure, close on flow" law. However, it does not consider real-time valve opening dynamics and therefore does not fully capture valve dysfunction, particularly where the dysfunction involves partial closure. This research describes an updated version of this previous closed-loop CVS model that includes the progressive opening of the mitral valve, and is defined over the full cardiac cycle.

**Results:**

Simulations of the cardiovascular system with healthy mitral valve are performed, and, the global hemodynamic behaviour is studied compared with previously validated results. The error between resulting pressure-volume (PV) loops of already validated CVS model and the new CVS model that includes the progressive opening of the mitral valve is assessed and remains within typical measurement error and variability. Simulations of ischemic mitral insufficiency are also performed. Pressure-Volume loops, transmitral flow evolution and mitral valve aperture area evolution follow reported measurements in shape, amplitude and trends.

**Conclusions:**

The resulting cardiovascular system model including mitral valve dynamics provides a foundation for clinical validation and the study of valvular dysfunction *in vivo*. The overall models and results could readily be generalised to other cardiac valves.

## Background

Mitral insufficiency (MI) is a frequent valvular pathology that develops as a result of dysfunction or modification in one of the elements of the mitral valvular complex (leaflets, tendinous chords, papillary muscles, left atrial wall or ventricular myocardium located next to papillary muscles). During MI, blood in the left ventricle flows back through the mitral valve towards the left atrium due to the loss of integrity of the valve, and a loss of the atrio-ventricular pressure gradient during systole [1-3]. The presence of a MI leads to a chronic overload in volume which is responsible for left ventricular dilatation, which, in its turn, increases the mitral insufficiency [3].

Ischemic mitral insufficiency (IMI) results from a ventricular remodeling usually following myocardial infarction. It is observed in 15 to 30% of the patients after acute myocardial infarction [4] and in 56% of the patients having a chronic left ventricular systolic dysfunction (ejection fraction < 40%) [5]. IMI is a dynamic condition whereby the severity of the regurgitation changes with time. This dynamic behavior can be responsible for the non-detection of the insufficiency for certain patients at high risk of morbidity and mortality [6,7]. Recent clinical studies have detected and quantified this dynamic behavior using Doppler echography [3].

As it is well-known mathematical models of the cardiovascular system (CVS) offer a promising method of assisting in understanding cardiovascular dysfunction, this research propose such a model to study IMI.

Mathematical models of the CVS vary significantly in their complexity and their objectives. They range from the simple Windkessel model [8], a zero-dimensional model, to very complex network representations of the vascular tree [9] or even finite element models of several million degrees of freedom [10,11]. All of them have different uses or goals, but they all share the common goal of understanding non invasively the cardiovascular function [12].

One example of low to intermediate complexity model, known as a "minimal cardiac model", has been developed and optimized [13-15] to assist health professionals in selecting reliable and appropriate therapies for Intensive Care Unit (ICU) patients. It is based on a "pressure-volume" (PV) lumped element modeling approach, the principle of which is that the continuous variation of the system's state variables in space is represented by a finite number of variables, defined at special points called nodes [12]. The cardiovascular system is thus divided into several chambers described by their own PV relationship [13,16]. The main advantage of this technique is that it only requires a small number of parameters, allowing for easy and rapid simulations and for patient-specific identification of disease state at the bedside with readily available clinical data [17-19].

This model has been proven to provide reliable description of the global cardiac function in several disease states such as pulmonary embolism and septic shock [20,21]. However, it does not allow for the description of lower anatomical scales, such as the valvular level.

The simplest description of the heart valve used in 0D CVS models represents the valve as a diode plus a resistance [9,16,22-24]. This description assumes the ideal characteristic of one-way flow in a heart valve, while more complex dynamics, such as regurgitant flow, can't be simulated. Žáček and Krause [25] used time dependent drag coefficients in a way that valve closing is achieved by letting the drag coefficient approach infinity. This model improves heart valve modelling, but the leaflet motion was prescribed instead of computed. An attempt to describe the progressive opening of the mitral valve based on physical properties of the valve was made by Szabó *et al*. [12,26-28]. However, their model is only valid during the early ventricular filling phase also referred to as E-wave filling. As we want to keep the minimal model's simplicity and therapeutic use, we can't use other valve models that are either too complex or/and do not account for dynamics over a full cardiac cycle [29-31].

This paper presents a minimal closed-loop model of the CVS including a description of the progressive opening and closing of the mitral valve at more refined scale. This new multi-scale model is validated for a healthy mitral valve. The impact of the progressive opening and closing of the mitral valve on circulatory dynamics is analyzed, with emphasis on IMI. Clinically, understanding this impact and the ability to identify it from clinical data would lead directly to new model-based diagnostic capabilities.

## Methods

This section first describes the existing and previously validated minimal model of the cardiovascular system, on which our model is based. Then, it describes a model of the mitral valve and its integration into the CSV model. Finally, methods used to analyse and criticize the results are presented.

### CVS model

**Figure 1:**
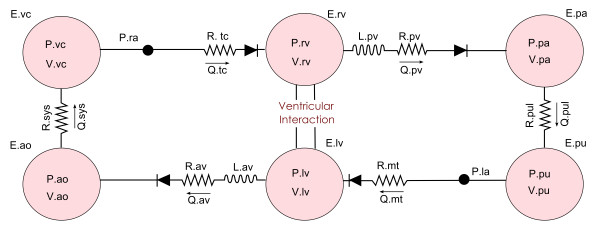
**Cardiovascular system model consisting of 6 cardiac chambers, vascular flow resistances, on/off heart valves and including ventricular interaction**. The 6 cardiac chambers are the left ventricle (lv), the right (rv) ventricle, the vena cava (vc), the aorta (ao), the pulmonary artery (pa) and the pulmonary veins (pu). Systemic and pulmonary circulation flow resistances are *R_sys _*and *R_pul_*. The cardiac valves are modeled by a resistance (*R_mt_*, *R_ao_*, *R_tc _*and *R_pv_*) and a diode. Inertia of the blood in the aorta and pulmonary artery are described by hydraulic inductances (*L_av _*and *L_pv_*, respectively).

The CVS model used to describe the cardiovascular system was first described by Smith et al [13] and has already been validated *in silico *and in several animal model studies [15,17,20,21,32,33]. It is a lumped parameter model consisting of six elastic chambers, the left ventricle (lv), the right (rv) ventricle, the vena cava (vc), the aorta (ao), the pulmonary artery (pa) and the pulmonary veins (pu), linked by vessels presenting a resistance to the blood flow. The diagram of this model is presented in Figure 1.

The systemic and pulmonary circulation networks are modelled by the resistances *R_sys _*and *R_pul_*, respectively, while the cardiac valves are modeled by a resistance and a diode that opens when the pressure upstream exceeds the pressure downstream and closes when the flow of blood is reversed. Inertia of the blood in the aorta and pulmonary artery are described by hydraulic inductances (*L_av _*and *L_pv_*, respectively) at the exit of the ventricles. The model takes into account ventricular interaction by means of the displacement of the septum, as it was found to have a significant impact on the cardiovascular system dynamics [13,34-36].

Each cardiac chamber is described by its pressure (P) -volume (V) relationship and provides volume and flow conditions around the physiological elements modelled by the resistances. Both cardiac chambers describing the ventricles are active. The relation linking the pressure to the volume is therefore not fixed and can be expressed by means of a time varying elastance (*E*(*t*)), an intrinsic property of each active cardiac chamber defined as:

(1) $$E\left(t\right)=\frac{P\left(t\right)}{V\left(t\right)}$$

**Figure 2:**
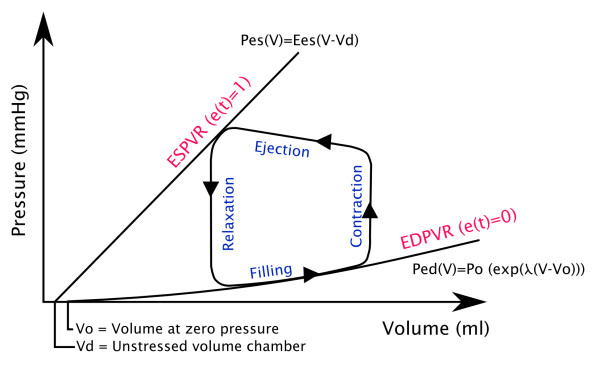
**Overview of a pressure-volume loop stuck between the pressure-volume relationships at the end of systole (ESPVR) and at the end of diastole (EDPVR)**.

This PV relationship varies between the PV relationships at the end of systole (ESPVR) and at the end of diastole (EDPVR), as shown on Figure 2.

(2) $$ESPVR:P_{ES}\left(V\right)=E_{ES}\left(V-V_{d}\right)$$

(3) $$EDPVR:P_{ED}\left(V\right)=P_{0}\left(e^{\lambda \left(V-V_{0}\right)}-1\right)$$

Where *P_ES _*is the end-systolic pressure, *E_ES _*the end-systolic elastance, *V *the volume, *V_d _*the volume at zero pressure, *P_ED _*the end-diastolic pressure and *P*_0_, *λ*, *V*_0 _are three parameters of the nonlinear relationship.

The transition between these two extremities is realised by several quasi-linear PV relationships, the slope of which is given by the activation function, also named the driver function, *e(t) *:

(4) $$e\left(t\right)=\overset{N}{\underset{i=1}{\sum }}A_{i}e^{-B_{i}{\left(\left(t\;mod\;D_{i}\right)-C_{i}\right)}^{2}}$$

With the number of Gaussians *N *= 1, the magnitude *A*_1 _= 1, the width *B*_1 _= 80*s*^-2 ^, the delay *C*_1 _= 0.27 *s *and the duration of a cardiac cycle *D*_1 _= 1*s*, as defined in Smith *et al*. [15]. Heart rate (*HR beats*/*min*) does not appear explicitly, but it is used to determine the value of one diver function parameter, *D*_1 _= 60/*HR*. This model doesn't use ECG data, but information relative to timing, delay and duration of the ventricular contraction is contained in the driver function parameters. This activation function accounts for myocardial activation and drives the changes in elastance. It varies between 0 and 1.

Then, the pressure in the ventricle at any time *t *of a cardiac cycle is linked to the volume by:

(5) $$\begin{array}{lll} P\left(t\right)=e\left(t\right)\times & E_{ES}\left(V\left(t\right)-V_{d}\right)\; & \text{(1)}\\ & +\left(1-e\left(t\right)\right)\; & \text{(2)}\\ & \times P_{0}\left(e^{\lambda \left(V\left(t\right)-V_{0}\right)}-1\right)\; & \text{(3)}\\ & \; & \text{(4)} \end{array}$$

The 4 elastic cardiac chambers representing the major blood vessels are passive and the pressure is assumed to be proportional to the volume of the chamber. The constant ratio of P/V is denoted E and represents the so-called elastance of the chamber.

The behaviour of each chamber is characterised by the flow in and out of the chamber (*Q_in_*, *Q_out_*), the pressure upstream and downstream (*P_up_*, *P_down_*), the resistances of the valves (*R_in_*, *R_out_*), and inertia of the blood (*L_in_*, *L_out_*) [13], yielding:

(6) $$\frac{dV}{dt}=Q_{in}-Q_{out}$$

(7) $$\frac{dQ_{in}}{dt}=\frac{P_{up}-P-Q_{in}R_{in}}{L_{in}}$$

(8) $$\frac{dQ_{out}}{dt}=\frac{P_{down}-P-Q_{out}R_{out}}{L_{out}}$$

Equations 6 and 7 are solved when *Q_in _*> 0, during the filling stage, and Equations 6 and 8 are solved when *Q_out _*> 0, during the ejection stage. During the iso-metric expansion and contraction phases, the model becomes much simpler, with volume and pressure linked by Equation 5.

Four cardiac valves are located in the heart, at the entrance and the exit of each ventricle. On Figure 1, these are represented by the electric symbol of a diode. Relations 7 and 8 describe the flows, but do not take into account the presence of valves regulating the flow. Indeed, they allow backflow (i.e. a negative blood flow) in the system. The physiological role of valves is exactly to prevent backflow by closing. To correctly model the effect of the valves, a negative flow must be replaced by no flow. It can be very simply modeled by replacing every appearance of a flow (*Q*) controlled by a valve by (*H(Q) Q*), where the notation *H *denotes the function of Heaviside [14], defined by:

(9) $$H\left(x\right)=\left\{\begin{array}{c} 0\;if\;x\leq 0\\ 1\;if\;x>0 \end{array}\right)$$

The introduction of this *H *function allows to describe the "close on flow" property [13,15] of the valve. To describe the "open on pressure" property [13,15], another *H *function must be introduced in Equations 7 and 8, to allow blood flow (*Q*) from the time when upstream pressure is higher than downstream pressure, until the time when blood flow becomes negative. It is written. *H*((*P_up _*- *P_down_*) + *Q *- 0.5).

Finally, the overall hemodynamic model reads the system of differential equations (Equations 10 to 19):

(10) $$\frac{dV_{pu}}{dt}=H\left(Q_{pul}\right)Q_{pul}-H\left(Q_{mt}\right)Q_{mt}$$

(11) $$\begin{array}{l} \frac{dQ_{mt}}{dt}=H(H(P_{pu}-P_{lv})+H(Q_{mt})\\ \;\;\;\;\;\;\;-0.5)\\ \;\;\;\;\;\;\;\times [(1/L_{mt})((P_{pu}-P_{lv})\\ \;\;\;\;\;\;\;-Q_{mt}R_{mt})] \end{array}$$

(12) $$\frac{dV_{lv}}{dt}=H\left(Q_{mt}\right)Q_{mt}-H\left(Q_{av}\right)Q_{av}$$

(13) $$\begin{array}{l} \frac{dQ_{av}}{dt}=H(H(P_{lv}-P_{ao})+H(Q_{av})\\ \;\;\;\;\;\;\;-0.5)\\ \;\;\;\;\;\;\;\times [(1/L_{av})(\left(P_{lv}-P_{ao}\right)\\ \;\;\;\;\;\;\;-Q_{av}R_{av})] \end{array}$$

(14) $$\frac{dV_{ao}}{dt}=H\left(Q_{av}\right)Q_{av}-H\left(Q_{sys}\right)Q_{sys}$$

(15) $$\frac{dV_{vc}}{dt}=H\left(Q_{sys}\right)Q_{sys}-H\left(Q_{tc}\right)Q_{tc}$$

(16) $$\begin{array}{l} \frac{dQ_{tc}}{dt}=H(H(P_{vc}-P_{rv})+H(Q_{tc})\\ \;\;\;\;\;\;\;-0.5)\\ \;\;\;\;\;\;\;\times [(1/L_{tc})(\left(P_{vc}-P_{rv}\right)\\ \;\;\;\;\;\;\;-Q_{tc}R_{tc})] \end{array}$$

(17) $$\frac{dV_{rv}}{dt}=H\left(Q_{tc}\right)Q_{tc}-H\left(Q_{pv}\right)Q_{pv}$$

(18) $$\begin{array}{l} \frac{dQ_{pv}}{dt}=H(H(P_{rv}-P_{pa})+H(Q_{pv})\\ \;\;\;\;\;\;\;-0.5)\\ \;\;\;\;\;\;\;\times [(1/L_{pv})(\left(P_{rv}-P_{pa}\right)\\ \;\;\;\;\;\;\;-Q_{pv}R_{pv})] \end{array}$$

(19) $$\frac{dV_{pa}}{dt}=H\left(Q_{pv}\right)Q_{pv}-H\left(Q_{pul}\right)Q_{pul}$$

### Mitral valve model

The main drawback of using the Heaviside functions to model the behaviour of the valves, is that this cannot take into account the physiological time scale of valve opening [37]. Therefore, the initial model introduced above is not able to fully capture valve dysfunctions.

**Figure 3:**
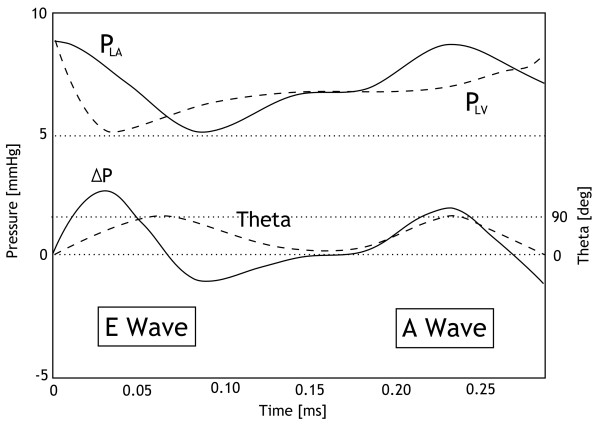
**Animal data **[37]**showing measured Pla, Plv and theta, and the calculated ΔP**. Pla and Plv profiles were measured invasively with catheters. This animal data also consisted of distance measurements of the amount the chords moved during a heartbeat, as measured by camera in a catheter or endoscope. It was assumed the movement of the chords would reasonably correlate to the change in opening angle, *θ(t)*.

As mentioned previously, an attempt to describe the progressive opening of the mitral valve was made by Szabó *et al*. [12,26-28] to assess diastolic left ventricular function based on Doppler velocity waveforms and cardiac geometry. The Szabó *et al*. model of early ventricular filling [12,28] begins at the time of mitral valve opening, when the pressures in the atrium and ventricle are equal and it describes the flow and pressure during ventricular filling until the atrial systole. The input pressure difference profile, *ΔP(t)*, shown in Figure 3 was taken from measured animal data [37]. This pressure difference was inferred from left atrial pressure (Pla) and left ventricular pressure (Plv) profiles, measured invasively with catheters. The two peaks in valve opening angle *θ(t) *evolution in Figure 3 refer to the E-wave and A-wave, which correspond to the passive filling of the ventricle and the active filling resulting from atrial contraction, respectively.

The main limitation of the mitral valve model by Szabó *et al*. is that it is only valid during a small part of the cardiac cycle (the E-wave). To couple the mitral valve model with the existing closed-loop CVS model, the model must be valid over a complete cardiac cycle. However, the existing CVS model does not include a chamber for the left atrium so it does not strictly capture atrial systole, also referred to as the A-wave [13]. In this research, the mitral valve dynamics during the active filling phase is assumed to be the same as during the passive filling phase so that all valvular parameters fixed by Szabo [12,28] are now used over the entire cardiac cycle. The A-wave is therefore not modelled.

As proposed by Szabo [12,28], a system of three ordinary differential equations can be used to describe the dynamics of the mitral valve. The first equation describes the flow rate through the mitral valve (*Q_mt_*). Thus, the corresponding equation from the original CSV model has been modified to add a term that accounts for the variation in the cross-sectional area of the mitral aperture. The differential equation then becomes:

(20) $$Q_{mt}^{\cdot }=\frac{P_{pu}-P_{lv}}{L_{mt}}-Q_{mt}\frac{R_{mt}}{L_{mt}}+Q_{mt}\frac{A\dot{(}t)}{A(t)}$$

Where:

*P_pu _*= pulmonary veins pressure

*P_lv _*= left ventricular pressure

*Q_mt _*= instantaneous flow rate through the mitral valve

*R_mt _*= mitral viscous resistance

*L_mt _*= mitral inertance term

*A *= area of mitral valve aperture

The resistance and the inertance of the mitral valve, *R_mt _*and *L_mt_*, are defined to take into account the existence of two states of the mitral valve (open and close). Therefore, the "open on pressure, close on flow" law is no longer required in the equation defining the dynamics of transmitral blood flow. Thus, the *H *(*H*(*P_pu _*- *P_lv_*) + *H *(*Q_mt_*) - 0.5) and *H *(*Q_mt_*) factors can be removed from Equation 10, 11 and 12.

When the valve is open, *R_mt_*, is low and when the valve is closed, the resistance is infinitely high. Applying the definition of hydraulic resistance in a cylindrical flow to this case [12] yields:

(21) $$R_{mt}=\frac{8\pi \mu l}{A{\left(t\right)}^{2}}$$

Similarly, the inertance is defined:

(22) $$L_{mt}=\frac{\rho l}{A\left(t\right)}$$

Where *ρ *represents blood density in *kg/m^3^*, *l *represents the blood column length through the mitral valve in *m *and *μ *the viscosity in *Ns/m^2^*.

The case *A*(*t*) → 0 is numerically prevented working with (*A*(*t*) + *ε*) instead of *A*(*t*) in the numerical simulations, when it is needed to divide by *A*(*t*).

### Dynamics of the mitral valve

The intrinsic dynamics of the valve must be able to describe all the modifications applied that are not due to an outside force. Therefore, it depends on the mechanical and geometrical properties of the valve, as well as on its composition. It is assumed that intrinsic dynamics is governed by inertia, due to the mass and the size of leaflets, by the elasticity of valvular tissues, and by the damping due to the blood surrounding the leaflets. Szabo *et al*. proposed that variation of the effective area of the mitral valve aperture can be defined by a second order differential equation [12,28] similar to that defining the evolution of a linear damped oscillator.

In the presence of external forces, the dynamics becomes that of a forced oscillator. These forces are mainly due to the pressure difference between both faces of the valve. This pressure force is applied on the valve surface across the flow. When the mitral valve closes, it places the surface (*A_max _*- *A*) across the fluid flow, where *A_max _*is the maximal surface that the mitral aperture can reach.

The second order differential equation defining area of mitral valve aperture is written:

(23) $$\begin{array}{l} \frac{1}{\omega^{2}}Ä+\frac{2D}{\omega }\dot{A}+A\\ =(A_{max}-A)\\ \times \left[K_{s}(P_{pu}-P_{lv})\right] \end{array}$$

where *D *is the damping coefficient describing the amount of damping experienced by the valve cusps, *ω *is the natural frequency of the valve, *A_max _*is the maximal surface that the mitral aperture can reach and *K_S _*is a coefficient introduced to adapt has the dimension of right term.

This approach introduces two new state variables, *A *and  Ȧ, and consequently two new ordinary differential Equations 24 and 25.

(24) $$\frac{dA}{dt}=Ȧ$$

(25) $$\begin{array}{lll} \frac{dȦ}{dt}=\omega^{2} & \left(A_{max}-A\right)\left[K_{s}\left(P_{pu}-P_{lv}\right)\right]\; & \text{(1)}\\ & \;-2DȦ\omega -A\omega^{2}\; & \text{(2)}\\ & \; & \text{(3)} \end{array}$$

To ensure that *A*(*t*) remains positive, the two differential Equations 24 and 25 are pre- multiplied by a Heaviside function: *H*{*H*(*P_pu _*- *P_lv_*) + *H*(*A*) - 0.5}. The differential equation is thus multiplied by zero when *P_pu _*<*P_lv _*and *A *becomes zero, and variations of *A *and  Ȧ will be allowed when *P_pu _*= *P_lv_*.

The resulting overall hemodynamic model reads now the system of differential equations (Equations 26 to 37):

(26) $$\frac{dV_{pu}}{dt}=H\left(Q_{pul}\right)Q_{pul}-Q_{mt}$$

(27) $$\begin{array}{l} \frac{dQ_{mt}}{dt}=[(1/L_{mt})((P_{pu}-P_{lv})\\ \;\;\;\;\;\;-Q_{mt}R_{mt})]\\ \;\;\;\;\;\;+\left(Q_{mt}A\right)/\dot{A} \end{array}$$

(28) $$\frac{dV_{lv}}{dt}=Q_{mt}-H\left(Q_{av}\right)Q_{av}$$

(29) $$\begin{array}{l} \frac{dQ_{av}}{dt}=H(H(P_{lv}-P_{ao})+H(Q_{av})\\ \;\;\;\;\;\;\;-0.5)\\ \;\;\;\;\;\;\;\times [(1/L_{av})((P_{lv}-P_{ao})\\ \;\;\;\;\;\;\;-Q_{av}R_{av})] \end{array}$$

(30) $$\frac{dV_{ao}}{dt}=H\left(Q_{av}\right)Q_{av}-H\left(Q_{sys}\right)Q_{sys}$$

(31) $$\frac{dV_{vc}}{dt}=H\left(Q_{sys}\right)Q_{sys}-H\left(Q_{tc}\right)Q_{tc}$$

(32) $$\begin{array}{l} \frac{dQ_{tc}}{dt}=H(H(P_{vc}-P_{rv})+H(Q_{tc})\\ \;\;\;\;\;\;\;-0.5)\\ \;\;\;\;\;\;\;\times [(1/L_{tc})((P_{vc}-P_{rv})\\ \;\;\;\;\;\;\;-Q_{tc}R_{tc})] \end{array}$$

(33) $$\frac{dV_{rv}}{dt}=H\left(Q_{tc}\right)Q_{tc}-H\left(Q_{pv}\right)Q_{pv}$$

(34) $$\begin{array}{l} \frac{dQ_{pv}}{dt}=H(H(P_{rv}-P_{pa})+H(Q_{pv})\\ \;\;\;\;\;\;\;-0.5)\\ \;\;\;\;\;\;\;\times [(1/L_{pv})((P_{rv}-P_{pa})\\ \;\;\;\;\;\;\;-Q_{pv}R_{pv})] \end{array}$$

(35) $$\frac{dV_{pa}}{dt}=H\left(Q_{pv}\right)Q_{pv}-H\left(Q_{pul}\right)Q_{pul}$$

(36) $$\frac{dA}{dt}=H\left(H\left(P_{pu}-P_{lv}\right)+H\left(A\right)-0.5\right)Ȧ$$

(37) $$\begin{array}{l} \frac{d\dot{A}}{dt}=H(H(P_{pu}-P_{lv})+H(A)-0.5)\\ \;\;\;\;\;\;\;\times [(A_{max}-A)\omega^{2}K_{s}(P_{pu}\\ \;\;\;\;\;\;\;-P_{lv})-2D\dot{A}\omega -A\omega^{2}] \end{array}$$

These differential equations are valid over the entire cardiac cycle. All other equations in the CVS model are unchanged.

Simulations of this resulting extended and physiologically multi-scale CVS model are performed using a standard ODE solver in Matlab (The MathWorks, USA). Note that this approach to describe the mitral valve can be generalized to other cardiac valves. The previously validated CVS model with Heaviside formulation and the new CVS model with variable mitral valve effective area are simulated for a healthy human using parameters from Tables 1,2,3 and 4.

**Table 1:** Base value of the pressure-volume relationship parameters used in the CSV model (healthy heart)

Parameter (unit)	Ees (mmHg/ml)	Vd (ml)	V0 (ml)	λ (1/ml)	P0 (mmHg)
Left ventricle free wall (lvf)	2.8798	0	0	0.033	0.1203

Right ventricle free wall (rvf)	0.5850	0	0	0.023	0.2157

Septum free wall (spt)	48.7540	2.00	2.00	0.435	1.1101

Pericardium (pcd)	-	-	200.00	0.030	0.5003

Vena cava (vc)	0.0059	0	-	-	-

Pulmonary artery (pa)	0.3690	0	-	-	-

Pulmonary vein (pu)	0.0073	0	-	-	-

Aorta (ao)	0.0	0	-	-	-

**Table 2:** Base value of the resistance and inertance parameters used in the CSV model

Parameter (unit)	R (mmHg s/ml)	L (mmHg s^2^/ml)
Mitral valve (mt)	0.0158	7.6968 × 10-5

Tricuspid valve (tc)	0.0237	8.0093 × 10-5

Aortic valve (av)	0.0180	1.2189 × 10-4

Pulmonary valve (pv)	0.0055	1.4868 × 10-4

**Table 3:** Base value of other parameters used in the CSV model

Parameter (unit)	Value (unit)
Heart rate (HR)	60 (bpm)

Total blood volume (Vtot)	5.5 (l)

Thoracic cavity pressure (Pth)	-4 (mmHg)

**Table 4:** Base value of parameters used in the mitral valve model (healthy mitral valve)

Parameter (unit)	Value (unit)
Static gain factor (Ks)	0.05 (1/mmHg)

Maximal mitral valve area (Amax)	1.1 (cm^2^)

Eigen frequency (ω)	30(rad/s)

Damping factor (D)	10 (1/rad)

### Mitral valve insufficiency

The closure and position of mitral leaflets are determined by the balance between two forces acting on them: the closing forces generated by the LV systolic contraction which effectively closes the valve, and the tethering forces that restrain the leaflets avoiding leaflet prolapse. When tethering forces are increased by displacement of the papillary muscles and the closure forces are reduced by LV dysfunction, the equilibrium between these two forces is broken in favor of tethering forces with displacement of the coaptation point of the leaflets in the ventricle, with a typical pattern of incomplete mitral leaflet closure.

A valve presenting a closure defect due to a restricted motion of the papillary muscles and whose leaflet structure is not affected, is considered to approach, at most, a case of ischemic mitral insufficiency (IMI). Thus, the size and the shape of the mitral leaflets do not change, and the parameters *ω *and *D *remain the same as in healthy simulations. In contrast, *Amax *is increased to take into account the mitral annular dilation [38] (Table 5). The closure defect (*dc*) observed consecutively to the displacement of the papillary muscles attached to the leaflets of the valve is taken into account in the Heaviside function that controls variations of *A *and  Ȧ. The differential equations related to *A *and  Ȧ (Equations 36 and 37) thus become:

**Table 5:** Base value of parameters used in the mitral valve model (mitral insufficiency)

Parameter (unit)	value
Static gain factor (Ks)	0.05 (1/mmHg)

Maximal mitral valve area (Amax)	1.3 (cm^2^)

Eigen frequency (ω)	30 (rad/s)

Damping factor (D)	10 (1/rad)

Defect of closure (dc)	0.2 (cm^2^)

(38) $$\begin{array}{l} \frac{dA}{dt}=H\{H(P_{pu}-P_{lv})+H(A-dc)-0.5\}\\ \;\;\;\;\;\;\;\times \dot{A} \end{array}$$

(39) $$\begin{array}{l} \frac{d\dot{A}}{dt}=H\{H(P_{pu}-P_{lv})+H(A-dc)-0.5\}\\ \;\;\;\;\;\;\;\times [\omega^{2}(A_{max}-A)K_{s}(P_{pu}\\ \;\;\;\;\;\;\;-P_{lv})-2D\dot{A}\omega -A\omega^{2}] \end{array}$$

## Results and discussion

Before discussing simulations of IMI, we find need to validate our model. Given the model assumptions, the main goal is to obtain a macroscopic behaviour similar to the existing, previously validated CVS model. PV loops will be compared to evaluate overall accuracy in pressure and volume at the end of systole and the end of diastole. A minimal error shows that the modified model matches the fundamental dynamics of the clinically validated original CVS model.

### Validation on normal human heart

The previously validated CVS model with Heaviside formulation and the new CVS model with variable mitral valve effective area are simulated for a healthy human using parameters from Tables 1,2,3 and 4.

**Figure 4:**
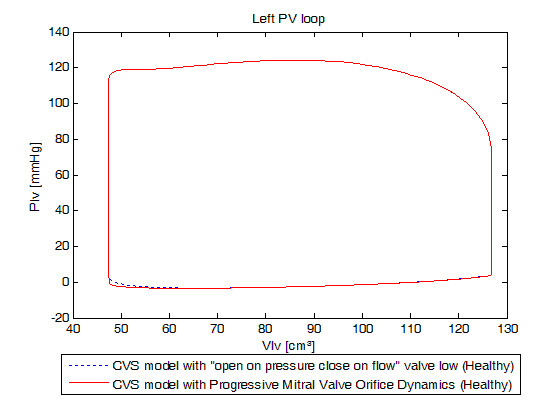
**Simulated left ventricular pressure-volume loops for a healthy valve.** Blue dotted: previously validated model, red linear: new model. Emphasis on the resemblance of the results in term of global behavior of the heart.

**Figure 5:**
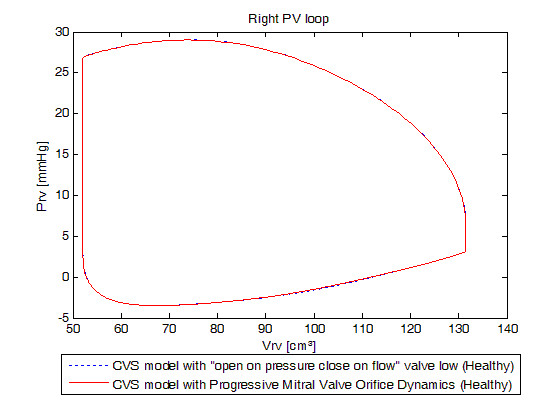
**Simulated right ventricular pressure-volume loops for a healthy valve.** Blue dotted: previously validated model, red linear: new model. Emphasis on the resemblance of the results in term of global behavior of the heart.

Figure 4 shows left ventricular PV loops and Figure 5, right ventricular PV loops of both models. Both models give effectively identical results. The mean relative error in PV loops at the four corners of the PV loops is (0 mmHg, 0.000 ml), (-0.01 mmHg, 1.176 ml), (0 mmHg, 0.130 ml) and (0 mmHg, 0 ml), which is within typical measurement error and variability. These errors may also be due to computational differences.

While showing no global cardiac function difference, the new model provides a more realistic description of the mitral valve. First, timing parameters are respected. In fact, physiologically, the passive filling of the left ventricle is characterised by 3 crossovers of pressures acting on both parts of the mitral valve. These 3 crossovers are critical for the timing of mitral valve opening. The first is related to the opening time of the mitral valve, the second to the maximum of the E-wave measured with Doppler and the last one to the end of the E-wave [39]. Time between the first and the third crossover is the duration of the early ventricular filling, typically between 0.14s and 0.20s depending of the heart state [12,37]. This period of early filling ends with the third crossover and is followed by a diastasis phase during which pressures on both parts of the valve remain very close, followed by atrial systole which leads to the active filling of the left ventricle, measured as the A-wave. The ventricle then starts to contract, driving ventricular pressure higher than atrial pressure, closing the mitral valve. Thus, the mitral valve remains open during about 0.27s to 0.32s [37].

**Figure 6:**
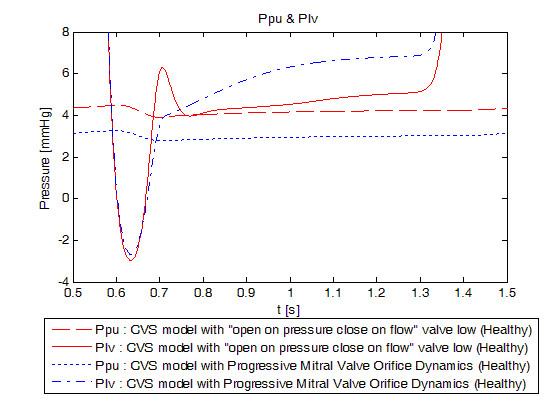
**Simulated pressure evolution on both sides of the mitral valve (Ppu and Plv) for a healthy valve**. Blue: previously validated model, red: new model. Emphasis on the 2 pressure crossovers.

**Figure 7:**
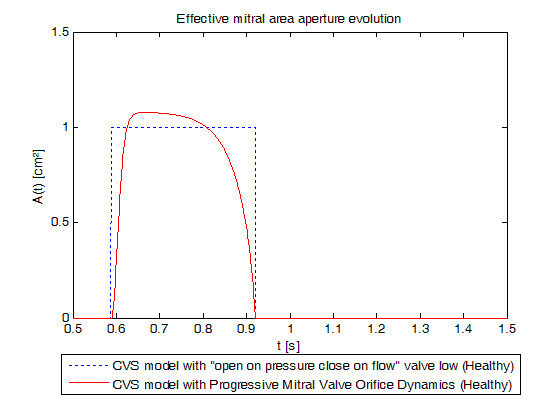
**Simulated mitral valve aperture area evolution for a healthy valve**. Blue dotted: previously validated model, red linear: new model. Emphasis on the differences between opening valve profiles.

Parameters of the mitral valve model have been chosen to fit opening and closing times. So even if all of pressure crossovers can't be found in the pressure evolution diagram (Figure 6), opening time, maximum opening of the early ventricular filling time and closing time are respected. In fact, mitral valve opens at time t = 0.589s, reaches its maximum at t = 0.666s and closes at time t = 0.921s as shown in Figure 7 which shows the evolution of mitral valve aperture area for the previous model with "open on pressure, close on flow" law for the mitral valve dynamics and for the new model. It means that mitral valve remains open during 0.3320s which is close to physiological values [37]. The time of maximum opening of the early ventricular filling is reached 0.077s after the mitral valve opens while this time is about 71 to 96 ms in literature [39], depending on the location of the measurement.

**Figure 8:**
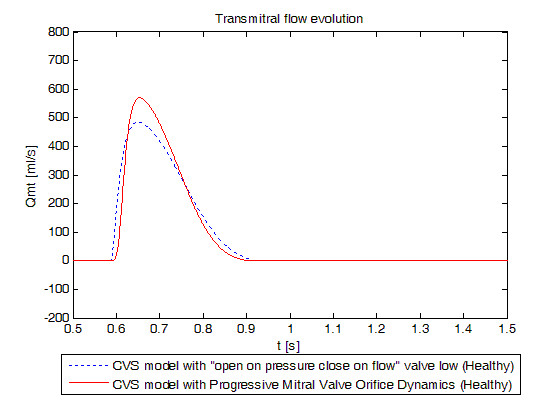
**Simulated transmitral flow evolution for a healthy valve**. Blue dotted: previously validated model, red linear: new model. Emphasis on the differences between flow profiles.

Figure 8 shows the evolution of transmitral blood flow during one cardiac cycle for the previous model with "open on pressure, close on flow" law for the mitral valve dynamics and for the new model. The maximum is reached at maximum mitral valve opening, and corresponds to a flow of 569.7 ml/s close to the 450 to 550 ml/s expected physiologically [40]. The value simulated is higher than the one found in literature because we only consider passive filling.

### Application to IMI

A physiologically evolution of mitral flow and mitral valve opening area during the early diastolic filling are needed to validate the updated model in dysfunction. In particular, the time of closure of the mitral valve is assessed to ensure proper closing time. Its change with dysfunction is also compared to clinical expectations. The specific dysfunction here modelled is mitral insufficiency consecutive to myocardial infarction and ventricular remodelling.

The loss of any portion of viable myocardium following myocardial infarction decreases the pumping ability of the heart, which is reflected in a deterioration of cardiac function. Postinfarction remodeling of the myocardium occurs to maintain cardiac output. These changes in the structure of the left ventricle alters the geometrical relationship between the ventricle and valve appartus generating a restricted leaflet motion termed incomplete mitral leaflet closure (IMLC) [38,41]. To model these changes, EESLVF (end systolic elastance of the left ventricle) is reduced by half, while VdLVF (dead volume of the left ventricle) and V0LVF (initial volume of the left ventricle) are first doubled and then gradually reduced to their initial values mimicking the experimental work of Shioura *et al*. [42].

**Figure 9:**
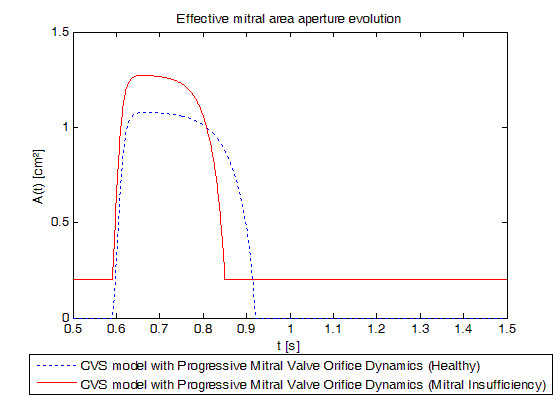
**Simulated mitral valve aperture area evolution for a healthy heart versus a remodelled heart with an ischemic mitral regurgitation**. Blue dotted: new model simulated for a healthy heart, red linear: new model simulated for a remodelled heart with an ischemic mitral regurgitation. Emphasis on the differences between healthy and diseased profiles, and the remaining opening of the diseased valve.

**Figure 10:**
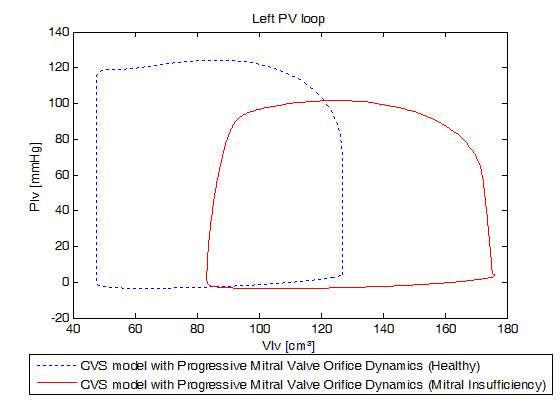
**Simulated left ventricular pressure-volume loops for a healthy heart versus a remodelled heart with an ischemic mitral regurgitation**. Blue dotted: new model simulated for a healthy heart, red linear: new model simulated for a remodelled heart with an ischemic mitral regurgitation. Emphasis on the differences of the results in term of global behaviour of the heart.

**Figure 11:**
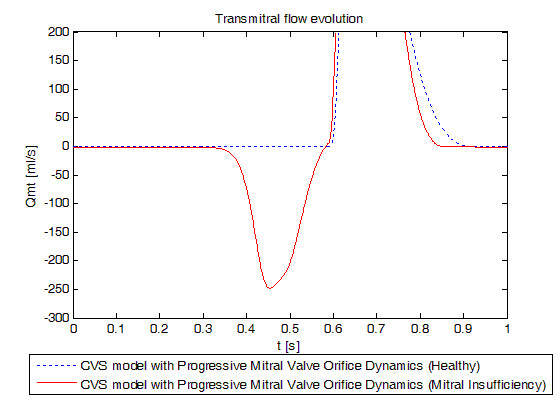
**Simulated transmitral flow evolution for a healthy heart versus a remodelled heart with an ischemic mitral regurgitation**. Red dotted: new model simulated for a healthy heart, blue linear: new model simulated for a remodelled heart with an ischemic mitral regurgitation. Emphasis on the backflow (negative flow) for the diseased profile.

Realistic acute mitral valve regurgitation due to a permanent defect of closure (Tables 2, 3, 5 and 6) was simulated. Figure 9 shows the evolution of mitral valve aperture area over a cardiac cycle evidencing the permanent opening of the mitral valve. Figure 10 shows the left PV loops for a healthy heart versus a remodelled heart with IMI. Left PV loops for the diseased heart are qualitatively similar to those observed experimentally [42] and clinically [1]. In fact, Raff and colleagues [1] have shown that pressure-volume loops are modified in the presence of valvular dysfunction and more specifically, that a global increase in ventricular volume is observed (PV loops move towards the right), as well as an increase in stroke volume. In Figure 10, minimal ventricular volume passes from 47.47 ml for the simulation of the healthy valve to 83.20 ml for the simulation of the incompetent valve while stroke volume passes from 79.43 ml to 92.80 ml. Raff et al. [1] also evidenced the disappearance of isovolumetric phases, isovolumetric contraction and isovolumetric relaxation. Figure 10 shows that these 2 phases have also disappeared because the mitral valve remains at least partly open during the entire cardiac cycle. In fact, when the ventricle contracts, some blood goes backward from the ventricle to the pulmonary veins chamber in the model resulting in negative transmitral flow, as shown in Figure 11, reducing the amount of blood at the end of the contraction just before the aortic valve opens.

**Table 6:** Base value of the pressure-volume relationship parameters used in the CSV model (heart remodeled after ischemic event)

Parameter (unit)	Ees (mmHg/ml)	Vd (ml)	V0 (ml)	λ (1/ml)	P0 (mmHg)
Left ventricle free wall (lvf)	1.4399	30	20	0.023	0.1203

Right ventricle free wall (rvf)	0.5850	0	0	0.023	0.2157

Septum free wall (spt)	48.7540	2.00	2.00	0.435	1.1101

Pericardium (pcd)	-	-	200.00	0.030	0.5003

Vena cava (vc)	0.0059	0	-	-	-

Pulmonary artery (pa)	0.3690	0	-	-	-

Pulmonary vein (pu)	0.0073	0	-	-	-

Aorta (ao)	0.0	0	-	-	-

Our results thus prove that our simple model can capture both the healthy state and valvular incompetence due to a defect in closure with appropriate variable changes.

### Limitations

While these results show strong physiological correlation to independently measured data, the second order model used to model the mitral valve aperture dynamics is not completely physiologically relevant. Specifically, the terms in the Equation 39 do not relate directly to the observed anatomical structure and function of the valve. Hence, the model itself, while capturing the effective dynamics, can provide no insight into specific disease impact or damage that results in valve dysfunction, even though it can model that dysfunction.

While we could compare pressure and flow curves, the measurement of the variable mitral valve area was not possible. To our best knowledge, no such data have been published for humans or pigs until recently. Using the newest developments in magnetic resonance technology and 4-dimensional echocardiography the authors are currently involved in a study to determine the time course of mitral valve opening. Even if we were not able to measure variable mitral valve area, the very good agreement between measured and calculated pressure and flow curves indicates that the simulated curves of mitral valve opening should be close to the natural time course.

In this model atrial contraction is not included. Further work will be needed to extend this model to include atrial systole and, to be able to model physiologically late diastolic filling. However, this limitation is not central to determine the efficiency of a refined mitral valve model, although some results in timing may be affected. This improvement would also widen the number of types of mitral insufficiencies that could be simulated. As noted before it would also enable a better assessment of timing problems due to a bad delay between atrial and ventricular contraction.

## Conclusions

This work describes a new multi-scale closed-loop physiological model of the cardiovascular system that accounts for progressive mitral valve motion. Simulations show that expected trends are respected for healthy and diseased valve states. These results suggest a further use of this model to track, diagnose and control valvular pathologies. The large number of valve model parameters indicates a need for new minimal and more physiologically relevant mitral valve models that are readily identifiable to achieve maximum benefit in real-time use of such models. However, the overall approach and modelling framework are readily generalisable to both other valves and more relevant valve models, proving the overall concept and approach.

## Competing interests

The authors declare that they have no competing interests.

## Authors' contributions

SP, TD, KTM, JGC and PCD conceived and developed the models and this analysis. SP did most of the computational analysis with input from TD, PCD, JGC and KTM. BL, MM, PL, VD, PK and LP supplied the clinical information and suggested how to model mitral valve dysfunctions. SP, TD, KTM, JGC drafted the manuscript primarily although all authors made contributions. All authors approved the final manuscript.
